# Personalized bone organoid using iPSC-derived cells for clinically relevant applications

**DOI:** 10.21203/rs.3.rs-5349885/v1

**Published:** 2024-12-13

**Authors:** Qi Gao, Victoria Teissier, Wenjuan Zhu, Meagan J Makarcyzk, Issei Shinohara, Masatoshi Murayama, Yosuke Susuki, Simon Kwoon-Ho Chow, Bruce A. Bunnell, Joy Wu, Hang Lin, Stuart B. Goodman

**Affiliations:** 1 Orthopaedic Research Laboratories, Department of Orthopaedic Surgery, Stanford University School of Medicine, Stanford, CA 94304, USA; 2 Stanford Cardiovascular Institute, Stanford University School of Medicine, Stanford, CA 94305, USA; 3 Center for Cellular and Molecular Engineering, Department of Orthopaedic Surgery, University of Pittsburgh School of Medicine, Pittsburgh, PA 15219, USA; 4 Department of Microbiology, Immunology and Genetics, University of North Texas Health Science Center, Fort Worth, TX 76107, USA; 5 Division of Endocrinology, Stanford University School of Medicine, Stanford, CA 94304, USA

**Keywords:** Stem cell, heterogeneity, personalized medicine, bone modeling

## Abstract

Patient-specific induced pluripotent stem cells (iPSCs)-based modeling potentially recapitulates the pathology and mechanisms more faithfully than cell line models and general animal models. Utilizing iPSC-derived cells for personalized bone formation research offers a powerful tool to better understand the role of individual differences in bone health and disease and provide more precise information for personalized bone regeneration therapies. Here we generated iPSC-derived mesenchymal progenitor cells (iMPCs), endothelial cells (iECs), and macrophages (iMØ), from different donors. Cellular markers, pluripotency properties, and immune regulatory properties were investigated. To replicate bone regeneration, we utilize different iPSC models and co-cultured three distinct cell types (iMPCs, iECs, and iMØ) in a 3D in vitro model derived from the same donor. Cells from different donors exhibited patient-specific characteristics and different regenerative capacities. Our study suggests that cells differentiated from iPSCs can be used to anticipate the effectiveness of cell-based therapies for personalized tissue regeneration.

## Introduction

Induced pluripotent stem cells (iPSCs) have been proposed for therapeutic regenerative purposes due to their potential to develop into different cell lineages. Importantly, modeling of various diseases using iPSC-derived cells can also be used to reduce the necessity for animal testing. Furthermore, since iPSCs are derived from patients’ own cells directly, iPSCs provide a more reliable source for precision personalized treatment^1 2 3^. Stable and robustly generated functional cells from iPSCs are crucial steps in utilizing iPSCs in regenerative medicine and drug discovery. Possible sources for heterogeneity among patients in the properties of iPSCs and iPSC-derived cells include cell proliferation, pluripotency, differentiation rate, and functional protein expression^4 5 6^. The cell heterogeneity observed in iPSCs and iPSC-derived cells mirrors individual variations and therefore holds promise for personalized medicine.

Bone regeneration is of great importance in clinical scenarios associated with trauma, fractures, infection, and other diseases. Autogenous bone graft is the most reliable and efficacious source but is limited in quality and quantity. The major cell population involved in bone regeneration is mesenchymal stem cells (MSCs), which have the capacity to differentiate into osteoblasts and contribute to the repair and regeneration of damaged bone. However, it is difficult to isolate sufficiently robust MSCs from elderly individuals and patients with chronic diseases, such as diabetes, kidney disease, and osteonecrosis^7 8^. iPSC-derived mesenchymal progenitor cells (iMPCs) are functionally similar to MSCs, can be used to investigate diverse MSC-derived subgroups, and have the potential for study of mechanisms of bone formation associated with specific patient variability. Endothelial cells (ECs) line blood vessels and are crucial for delivering nutrients, maintaining homeostasis, and pathologic angiogenesis. The functions of ECs are also highly related to their source and local microenvironment^9 10^. iPSC-derived ECs play a crucial role in studying disease states and engineering blood vessel grafts. Immune cells such as macrophages (MØ) are associated with tissue development, repair, and regeneration, and are important to pathological inflammation^11 12^. From this perspective, we compared the functions of iPSC-derived cells, including iMPCs, iECs, and iMØ. Four sources of iPSCs from four different donors of different ages and health status were used to explore the functional heterogeneity and potential therapeutic application in preclinical disease modeling. Modeling clinical pathology using iPSC-derived cells from autologous sources facilitates an understanding of individual biological mechanisms in disease and offers great potential for personalized interventions including more precise drug screening.

As the requirement for personalized medicine increases, the potential for iPSC-derived models is expanding. Employing cell coculture systems is a promising platform to mimic cellular interactions, tissue organization, and disease conditions in a more efficient and cost-effective way^13 14^. In the present study, we first generated key iPSC-derived cells and compared their phenotypes and function. The different capacities of differentiation into specific cell lineages were then investigated. Currently, micro physiological systems mimicking key physiological functions can be closely correlated with the properties of adult tissues. Here, we cocultured various iPSC-derived cell types in a 3D scaffold to mimic bone regeneration in clinical scenarios. We hypothesized that iMPSc, iECs, and iMØs would display heterogeneous phenotypes that are dependent on the host’s biological capacities. We examined the bone regeneration potential and the cellular response to inflammatory stimuli using iPSC-derived bone organoids. The research findings suggest differences in bone regeneration using iPSC-derived cells among different patients.

## Results

### iMPCs shared many similarities but they also showed different trilineage differentiable abilities.

**Fig 2.**
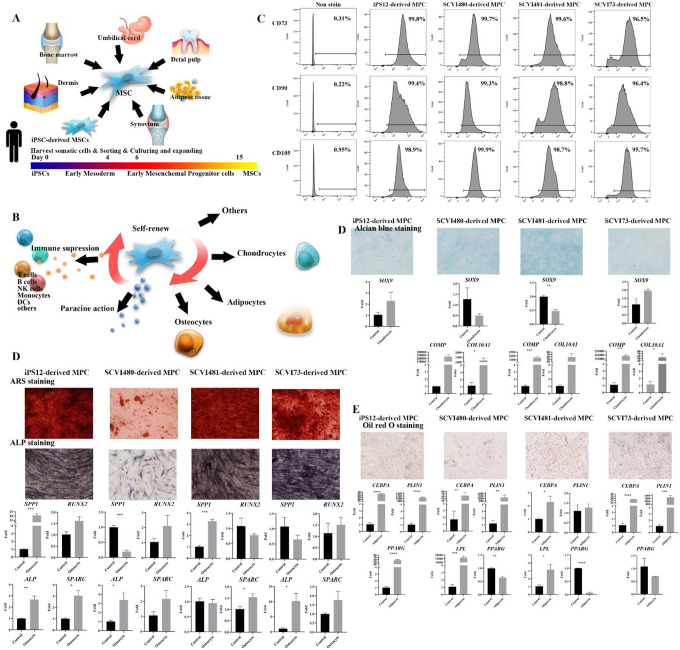
Generation and characterization of iPSC-derived MSCs. (A) Sources of MSCs. (B) Functions of MSCs. (C) Flow cytometric analysis of iPSC-derived MSC markers CD73, CD90, and CD105. (D-F) Trilineage differentiation of iPSC-derived MSCs. (D) Osteogenic differentiation visualized by Alizarin red staining, ALP staining, and qPCR analysis of *SPP1*, *RUNX2*, *ALP*, and *SPARC*. (E) Chondrogenic differentiation visualized by Alcian blue staining, and qPCR analysis of *SOX9*, *COMP*, and *COL10A1*. (F) Adipogenesis differentiation visualized by Oil red O staining, and qPCR analysis of *CEBPA*, *PLIN1*, and *PPARG*. (* *p* < 0.05, ** *p* < 0.01, *** *p* < 0.001, **** *p* < 0.0001.)

During the iPSC differentiation process, we were able to generate early mesodermal progenitor cells, followed by early mesenchymal progenitor cells, and finally, iMPCs. Primary MSCs typically exhibit the expression of CD105, CD73, and CD90, while lacking the expression of CD45 and CD34^15^. We checked these markers using flow cytometry. The differentiation efficiency of MPCs from all four donor iPSCs were higher than 95% as shown in Figure 2C, and the differentiated cells were suitable for further experiments without a specific sorting process.

MSCs, also known as mesenchymal stromal cells, possess remarkable trilineage differentiation ability, including osteoblasts (bone cells), chondrocytes (cartilage cells), and adipocytes (fat cells). Primary MSCs from different tissue sources show variable tendencies to differentiate into osteoblasts, chondrocytes, and adipocytes^16^. iPSC-derived MSCs are from different donors and sources, which can result in variations in their differentiation potentials. We tested the trilineage differentiation capacity of iMPCs in a 2D culture system. Osteogenesis markers (*RUNX2*, *OPN* (*osteopontin*)*, ALP*, and *SPP1* (*secreted phosphoprotein 1*)), chondrogenesis indicators (*COMP*, *SOX9*, and *COL10A1*), and representative adipogenesis genes (*CEBPA*, *LPL*, *PLIN1*, and *PPARG*) were quantified using qPCR. iMPCs exhibit varying differentiation potentials. Greater osteogenic and adipogenic tendency of iMPCs from the iPS12 cell line was observed, whereas there is lower ability to differentiation toward chondrocytes. SCVI480-derived iMPCs had superior adipogenic capacity but less osteopontin expression during osteogenesis process. iMPCs derived from SCVI3C1 cells had the highest potential for chondrogenic and adipogenic differentiation. We also verified the ability for osteogenesis by alkaline phosphatase (ALP) and Alizarin Red (AR) staining. Chondrogenesis and adipogenesis were confirmed using Alcian blue stain, and Oil red O staining, respectively. Results are shown in Figure 2D–F; there was no significant difference among these iMPCs in the above properties. Our results suggested that although iMPCs from different iPSC cell lines were found to share many characteristics including trilineage differentiation properties and surface markers, differences in gene expression profiles were detected that may impact the regenerative potential.

**Fig 3.**
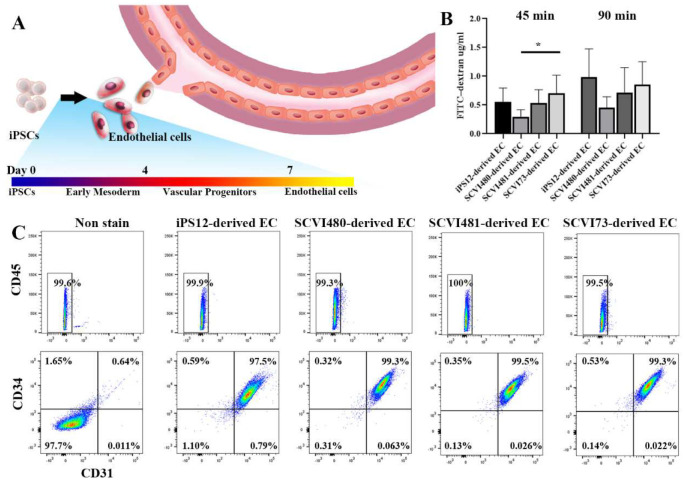
Generation and characterization of iPS-derived endothelial cells. (A) Endothelial cells line the blood vessel. (B) Measurement of the transcellular permeability of iPSC-derived ECs. (C) Flow cytometric analysis of EC markers CD31 and CD45. (* *p* < 0.05, ** *p* < 0.01, *** *p* < 0.001, **** *p* < 0.0001.)

We differentiated iPSCs to iECs, and CD34-positive cells were isolated and defined as endothelial cells. As shown in Figure 3, more than 97% of cells were positive for CD31 and CD34 after sorting, and negative for CD45. The major functions of ECs are barrier properties and angiogenic potential. To explore the barrier properties, iPSC-derived ECs were cultured in a transwell plate, and FITC-dextran permeability studies were conducted. The translocation of FITC-dextran was monitored. There were minor differences in the FITC–dextran flux in the transwell assay across the EC barrier, which indicates sufficient protection from leakage in all groups. The results supported the idea that iPSC-derived ECs can be used for vascular development without significant heterogeneity in these 4 donors.

### iMØ demonstrated comparable polarization characteristics.

**Fig 4.**
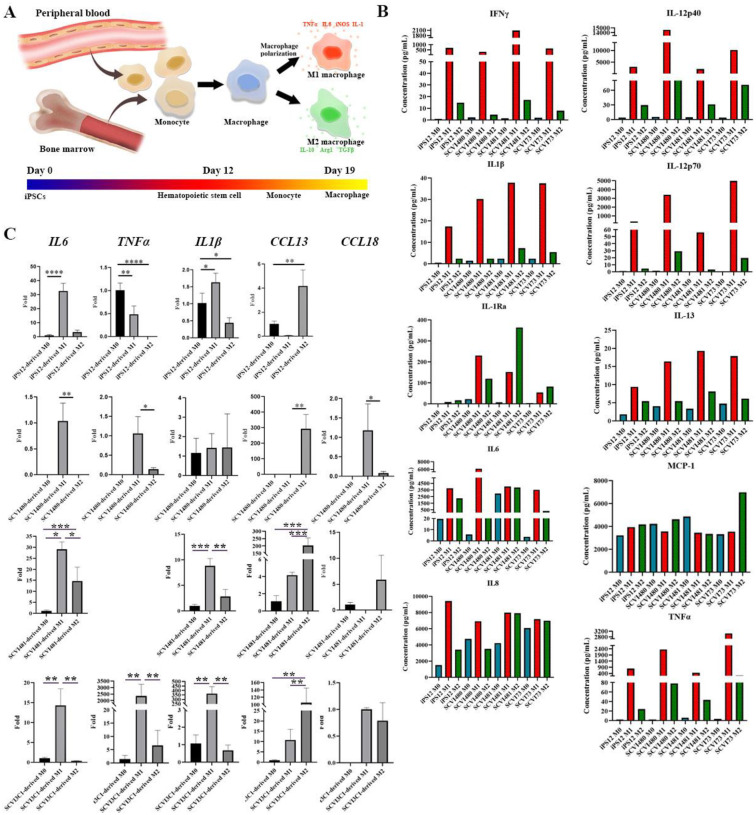
Comparison among macrophages generated from different iPSC cell lines. (A) Monocyte differentiation and macrophage polarization. (B) Cytokine secretion of naïve macrophages and polarized macrophages. (C) Gene expressions of inflammatory-related cytokines were quantified by qPCR. (* *p* < 0.05, ** *p* < 0.01, *** *p* < 0.001, **** *p* < 0.0001.)

Starting from the pluripotent state of iPSCs, we induced hematopoietic stem cells (HSCs) and subsequent CD34-positive HSCs were derived to macrophages by supplementing the medium with macrophage colony-stimulating factor (MCSF). Naïve iMØ respond to environmental stimuli and subsequently polarize into different phenotypes, which are often broadly characterized as pro-inflammatory (M1) or anti-inflammatory (M2) macrophages. We further assessed whether the iMØ faithfully recapitulated macrophage polarization. Luminex assay and qPCR were performed to compare cytokines and genes expressions between naïve iMØ and polarized iMØ. The iPSC-derived M1 macrophages demonstrated a higher secretion of inflammatory-related cytokines, such as IFNγ, IL-1β, and IL-6, in comparison to the iM0 and iM2 macrophages as shown in Figure 4B. The concentration differences among various donors were also observed. It has been suggested that inter-donor variation and the donor’s biological characteristics affect the therapeutic use of macrophages^18^. Our results from iPSC-derived macrophages align with the cytokine profile observed in primary macrophages.

When comparing gene expression, there was increased expression of M1 genes, *IL-6* and *IL-1β* in all the M1 iMØs when compared with M0 iMØs and M1 iMØs. *TNFα* gene expression varies in different groups. The expression levels of *TNFα* are elevated in M1 iMØs derived from SCVI480 and in iMØs derived from SCVI3C1. In contrast, TNFα is undetectable in macrophages derived from SCVI481, and its expression is notably reduced in iMØs derived from iPSC12. Expression of *CCL13* was significantly higher in all the M2 iMØ. Through an evaluation of the functions and applicability of iMØ in disease modeling, it was determined that iMØ exhibited a high degree of similarity to primary macrophages, despite the observed viability.

### Cellular crosstalk among iPSC-derived cells

**Fig 5.**
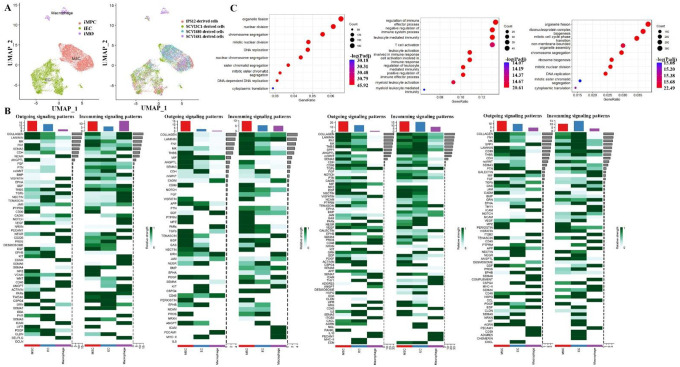
(A) UMAP projection of all iPSC-derived cells. (B) Cellular communications among the iPSC-derived cells. Heatmaps of the outgoing and incoming signaling pattern in each cell type. Cell types are distributed on the horizontal axis and signaling pathway on the vertical axis. The upper and right columns are the relative strength of the vertical and horizontal axes, respectively. (C) GO enrichment analysis of biological processes in iMPC, iEC, and iMØ.

Cells communicate by sending and receiving signals in their immediate surroundings. A network for cell-to-cell communication has been predicted using CellChat, linking a range of cell types relevant to bone including iMPCs, iMØ, and iECs through the monitoring of their interactions. We first annotated each cell types by interrogating the expression of known marker genes, as identified by differential gene expression (DGE) analysis. Uniform manifold approximation and projection (UMAP) dimensionality reduction analysis was applied to identify the marker genes for each cell type. Cell-type distributions in the four iPSC-derived cell lines are shown in Figure 5A–B. The marker gene expression of the cell types was *CD105* and *CD90* for iMPC, *ADGRL*, *SOX17*, *ECSCR*, *FLT1*, and *KDR* for iECs, *CD14*, *CD68*, *CD86*, and *CD44* for iMØ, respectively.

Heat maps were generated to provide a visual representation of the transmission of signals between cell types as shown in Figure 5B. Collagen, Laminin, and FN1(Fibronectin 1) signaling pathways are the major outgoing pathways in all these four iMPCs. Communication facilitated by ligand-receptor interactions between diverse cell types is essential for bone regeneration. We hypothesized that iMPCs secrete extracellular matrices, such as collagen, laminin, and FN1 to interact with other cells via adhesion molecules which in turn transduce inner cellular signals for cell growth and differentiation. Indeed, collagen and laminin have been shown to be linked with tissue repair. FN1 promotes chondrocyte differentiation and collagen production via TGF-β/PI3K/Akt pathway in mice with femoral fracture. FN1 overexpression promotes collagen production through the TGF-β/PI3K/Akt pathway^19^. In the iPS12-MPCs group, the outgoing of Wnt pathway was detected. Activation of the Wnt signaling pathway promotes stem cell growth, survival, and differentiation^20 21^. Furthermore, the MIF (Macrophage migration inhibitory) pathway was detected from the SCVI481 and SCVI3C1 groups, suggesting that coordinated crosstalk of MØs and MSCs plays a key role on bone regeneration. The TGFβ pathway was mainly noted in the SCVI480-MPCs and SCVI3C1-MPCs but not SCVI480-iMØ and SCVI3C1-iMØ. However, TGFβ pathway is a main outgoing pathway of iMØ in the other two groups. This indicates that the high expression of the same molecule may be due to different cell types. For iECs, the outgoing of IL-6 signaling was detected from SCVI481-ECs but not in other groups. SCVI481-ECs and SCVI3C1-ECs showed highly outgoing CXCL signaling pathway. Our results support the concept that iMØ, iMSCs, and iECs cooperate in the bone regeneration, and the cell-to-cell communication varies among different patients.

We also identified MØ-mediated mechanisms of osteogenesis and angiogenesis by examining signaling changes associated with MØ. We found that each iMØ cell lines exhibited a unique outgoing pathway with CD46 could be highly secreted by almost all iMØs. In the iPS12-MØ group, the outgoing pathway include GDF (growth differentiation factor) pathway, which may activate intracellular PI3K/Akt signaling cascade^22^. Outgoing of PROS (PIK3CA-related overgrowth spectrum) pathway also has the potential to hyperactivate the PI3K pathway. In SCVI480-MØ, outgoing signaling pathways, GDF and GRN, PECAM1 (platelet and endothelial cell adhesion molecule 1) suggested suppression M1 toward polarization and results a decreased synthesis of TNF^23 24 25^. The higher IL-10 pathway was detected from SCVI481-MØ group supports an anti-inflammatory potential. Outgoing of ADGRE5 (adhesion G protein-coupled receptor E5) and PECAM1 are highlighted in SCVI481-MØ and SCVI3C1-MØ, which indicate the potential inhibition of LPS induced NF-κB activation^25 26^. By examining the inferred cell–cell communication network in each iPSC group, we found that the activation of signaling pathways varies across different iPSC groups and types of cells.

To further explore the transcriptome features of each cell type, an examination of the enrichment of gene functions and signaling pathways within each cluster was carried out. As shown in Figure 5C, the biological function of iMPCs included “organelle fission”, “nuclear division”, and ‘chromosome segregation”. The biological function of iECs was related to “organelle fission”, “ribonucleoprotein complex biogenesis”, and “mitotic cell cycle phase transition”. The biological function of iMØs was connected to “regulation of immune effector process”, “negative regulation of immune system process”, and “leukocyte mediated immunity”.

### Personalized bone organoid using iPSC-derived cells

**Fig 6.**
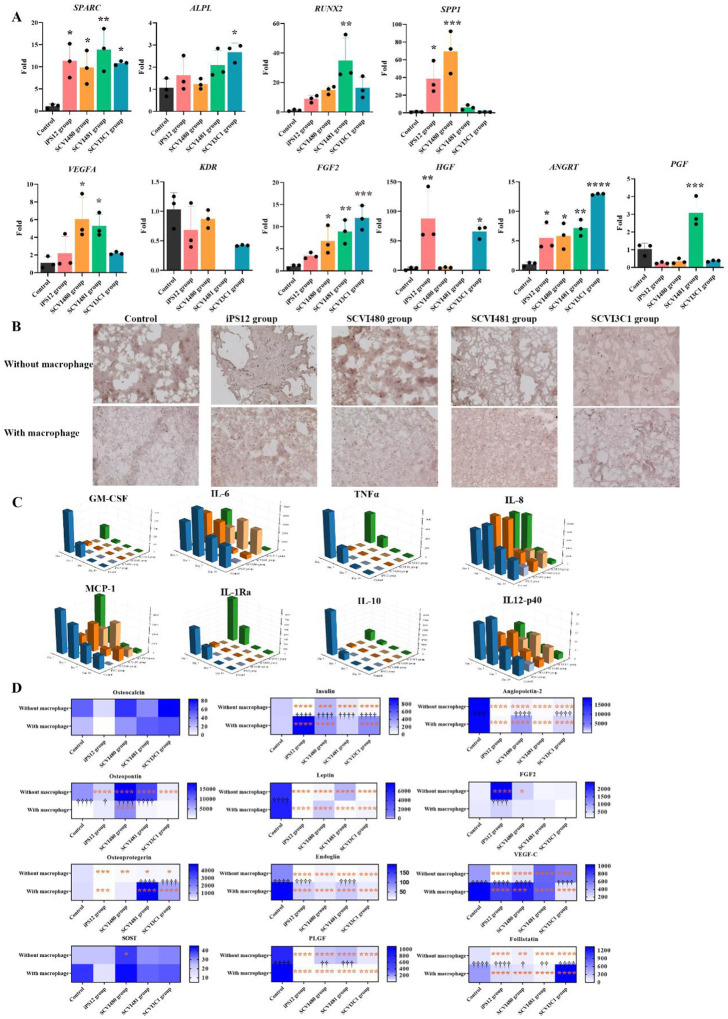
MØ regulate osteogenesis and angiogenesis. (A) Osteogenic and angiogenic markers were verified by qPCR. (B) ARS staining of the iPSC-bone organoid. (C) Luminex analysis was applied to quantify the cytokine changes in the supernatant. (D) Levels of osteogenesis and angiogenesis related factors with or without MØ at week 4. By comparing each iPSC group with the control group, the statistical result was denoted by *. The symbol † indicates the different between each group with or without MØ. (* *p* < 0.05, ** *p* < 0.01, *** *p* < 0.001, **** *p* < 0.0001. † *p* < 0.05, †† *p* < 0.01, ††† *p* < 0.001, †††† *p* < 0.0001)

Coculturing cells that differentiate from patient-specific iPSCs has utility as a disease model for developing patient-specific therapies. We first introduced iMPCs together with iECs derived from the same iPSC cell line into a 3D GelMA culture system to establish a personalized platform for precision bone regeneration. The coculture of primary MSC and HUVECs (human umbilical vein endothelial cells) was used as a control group. After 4 weeks of differentiation, expression levels of osteogenic genes, including *ALP*, *RUNX2*, *OPN*, and *SPP1*, and angiogenic genes, including *HGF*, *ANGRT*, *FGF2*, *KDR*, *PGF*, and *VEGFA*, were quantified using qPCR. *SPARC* and *ANGRT* were up-regulated in all the iPSC groups than the control group. Each iPSC group exhibited unique marker overexpression, suggesting distinct molecular expression within each group. These differences may reflect underlying variations in the osteogenesis and angiogenesis process, impacting the design and treatment for downstream applications in disease modeling and regenerative medicine. Osteogenic and angiogenic markers secreted to the cultured medium at the fourth week were quantified by Luminex and the results are shown in Figure 6D. All iPSC groups exhibited reduced levels of osteoprotegerin, leptin, endoglin, PLGF, and angiopoietin-2 compared to the control group. This indicates a decreased potential for osteogenesis and angiogenesis in the iPSC models.

Following our previous findings which indicated the necessity of MØ in bone regeneration^27^, we incorporated iMØ into our system to study the effects of MØ on osteogenesis and angiogenesis. We found that the secretion of osteoprotegerin significantly increased in the SCVI481 and SCVI3C1 groups compared to the control group. The secretion of osteopontin is higher in SCVI480 group than control group. Nevertheless, the angiogenesis potential in the iPSC groups was found to be lower than that of the control group.

Osteogenesis was further assessed by examining matrix calcification with Alizarin Red staining (ARS) at week 4. The MØ co-culture scaffolds had the highest calcium deposition and matrix mineralization compared with the other groups that did not include macrophages, suggesting a potential enhancement effect of macrophages on early bone formation.

Macrophages secrete inflammatory cytokines that regulate tissue homeostasis. The inflammatory cytokines were monitored, and the results are summarized in Figure 6C. In general, there was a consistent downward trend over time, implying a commonality in the decline observed among the groups except SCVI481 group. In the SCVI481 group, the levels of IL-6, MCP-1, and IL12-p40 showed a slow upward trend followed by a subsequent decline. GM-CSF, IL-10, IL1-Ra, and TNFα were highly expressed in the SCVI3C1 group and control group at day 1 while the expression levels in the other group were notably lower. Highly expression of IL-6, MCP-1, IL12-P40, and IL-8 at day 1 indicating an inflammatory environment.

### Different regenerative mechanisms in personalized bone organoids.

**Fig 7.**
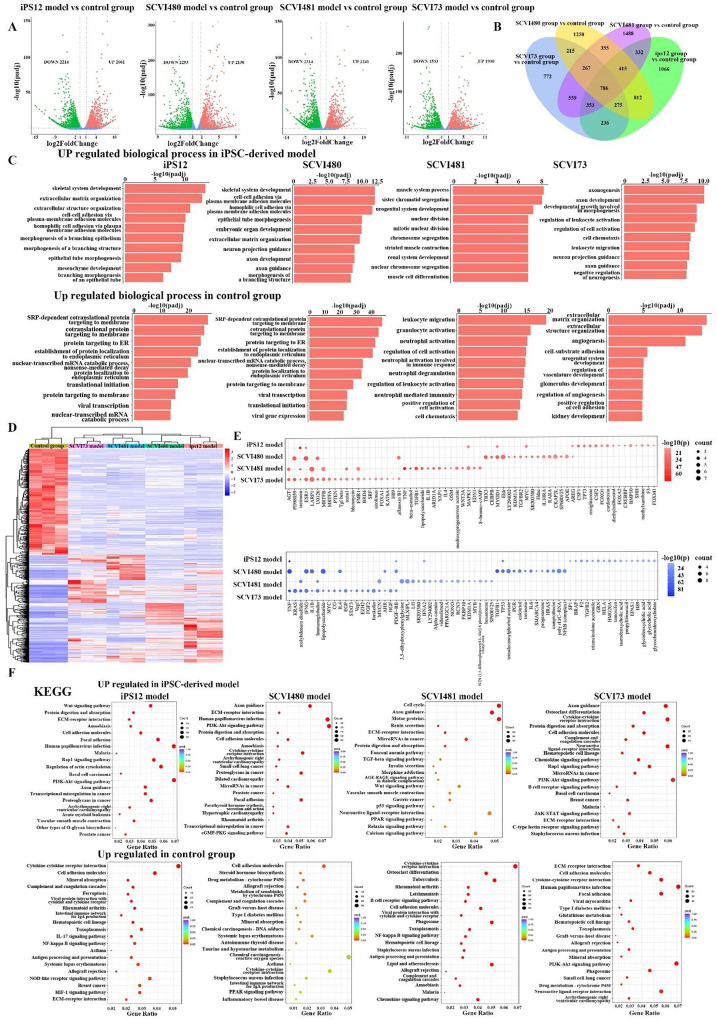
Volcano plots illustrating the DEGs between the iPSC groups and the control group. (B) Venn diagrams representing the differentially expressed genes specific or common in the coculture group. (C) GO functional analysis of DEGs between the iPSC groups and the control group. (D) The heatmap shows the top differentially expressed genes. (E) Predicted activators (red) and inhibitors (blue) in each iPSC group. (F) KEGG analysis of DEGs between the iPSC groups and the control group.

We conducted bulk RNA-seq analysis to explore the various molecular mechanisms in different cocultured iPSC groups. We determined differential gene expressions (DEGs) between control group and iPSC groups. A Venn diagram showed that 786 co-expression DEGs among the five groups. Compared to the control group, each iPSC group demonstrating up-regulated and down-regulated DEGs are shown in the volcano plot in Figure 7A. Gene oncology (GO) analyses of the DEGs were performed. Variances among iPSC groups suggest unique individual differences. The upregulated DEGs in the iPS12 group and SCVI480 group were mainly enriched for the biological process (BP) terms “skeletal system development”, “extracellular matrix organization” and “cell-cell adhesion”, while downregulated DEGs were significantly associated with “SRP-dependent cotranslational protein targeting to membrane”, and “protein targeting to ER”. For the SCVI481 group, “muscle system process” and “cell proliferation related processes” were highly activated, while “leukocyte migration” and “neutrophil activation” were down regulated. It indicates a decreased immunoregulation in the SCVI481 group. The SCVI3C1 group exhibited significant activation of “axonogenesis and regulation of leukocyte”, whereas “extracellular matrix organization and angiogenesis”, and “cell-substrate adhesion” were observed to be suppressed.

To identify downstream pathways, we analyzed the Kyoto Encyclopedia of Genes and Genomes (KEGG) pathways of DEGs in iPSC groups. The KEGG pathway analysis results showed that the upregulated DEGs in iPS12 and SCVI480 group were associated with “Wnt signaling pathway”, “ECM-receptor interaction”, “PI3K-Akt signaling pathway” (Figure 7F). Inflammation related pathways were downregulated in the iPS12 group, including “IL-17”, “NF-kappa B signaling”, and “antigen processing and presentation”. This result is consistent with the cell-to-cell communication analysis in the previous part that Wnt signaling pathway is highly upregulated in the iPS12 model. Furthermore, the decreased activation of inflammatory pathways aligns with the reduced expression of cytokines, which is in accordance with the low cytokine expression.

Ingenuity Pathway Analysis (IPA) was applied to predict activated and inhibited factors that regulate up-regulated and down-regulated genes observed. One mediator may produce opposite stimulating effects in different groups. For instance, the PI3K inhibitor LY294002 is an activator in the SCVI480 group but is an inhibitor in the SCVI481 group. IL-4 works as an activator within the SCVI481 group, while acting as a suppressor in both the SCVI480 and SCVI3C1 groups. Our findings indicate that the pathways involved in bone regeneration are closely associated with the cellular functionalities within the tissue. The prediction of varying reactions to the same mediators among different individuals highlights the significance in precision medicine.

## Discussion

### Functional heterogeneous in iPSC-derived cells

The selection of a proper cell source helps precision medicine and maximizes the therapeutic effect for specific individuals. Although iPSCs share a similar undifferentiated stem cell phenotype, heterogeneity has been reported in many studies, such as differentiation propensity and self-renew rate^4 28^. These differences impact the function of the differentiated cells and influence subsequent modeling applications. As multipotent stromal cells, MSCs can be differentiated into a variety of cell types including osteoblast, chondroblast, and adipocyte in vitro. Other promising features of MSCs include self-renewal capacity and immunomodulation via the secretion of various mediators^29 30^. These features hold great promise for tissue repair in the clinic. Heterogeneity exists in stem cell function of MSCs due to donor, tissue, and maintenance methods. The variable sources, potential for proliferation, differentiation, and metabolic, and immune regulation properties make it difficult to compare clinical trials. For example, MSCs from synovial are more likely to be differentiated into chondrocytes than MSCs from synovial fluid^31^. Bone marrow-MSCs (BM-MSCs) possess increased osteogenic and decreased adipogenic differentiation potentials than adipose tissue-derived MSCs (AD-MSCs)^32^. BM-MSCs and MSCs derived from umbilical cords (UC-MSCs) share similar immunosuppressive effects^33^, while donor sex is more likely to impact immune modulation^34^. Different transcriptional profiles were reported among MSCs from different tissues that may impact their function^35^. MSCs obtained from some patients did not show the normal differentiation capacity and therefore might be excluded for therapeutic purposes^36^. In addition, synovial MSCs from OA patients displayed enhanced osteogenic and adipogenic potentials, whereas those from patients with femoral-acetabular impingement syndrome exhibited greater chondrogenic potential^37^. In our study, we investigated the multifunctional activities of iMPCs, and found that although the surface markers are comparable, iMPCs derived from different iPSCs exhibited distinct trilineage differentiation characteristics. Further consideration should be given to the effects of these differentiation variances in cell coculture utilization and regeneration modeling.

Currently, the majority of reported experiments use MØs derived from peripheral blood mononuclear cells (PBMCs) or cell lines such as THP-1^38^. Here we use iPSCs as the major source of MØ. MØ polarizes to different phenotypes and plays important roles in immunomodulation, homeostasis, resolution of inflammation, and antigen presentation^39 40^. In vivo, abundant monocyte-derived MØs are recruited to the tissue under infection or inflammation. Upon stimulation, MØs are polarized to different functionally heterogeneous groups. In response to Toll-like receptors and IFN-γ, M1 phenotype MØs are obtained with intrinsic phagocytosis and pro-inflammatory properties. Contrastingly, MØs undergo an anti-inflammatory M2 phenotype when treated by IL-4 or IL-13. Polarization of MØs into either the M1 or M2 phenotype has become a promising therapeutic approach for dealing with inflammatory diseases. MØ heterogeneity and inter-donor functional differences have been widely reported^41^. The physical condition also impacts MØ heterogeneity which plays a critical role in regulating diseases^42^. Here we investigated the difference among iMØ. A comparable polarization feature has been observed, however, the levels of cytokines differ across various cell lines, resembling primary macrophages. The cytokine concentrations may also be altered by other cells in the subsequent co-culture experiments

### Personalized bone regeneration

In intramembranous ossification, bone tissue is directly synthesized by osteoblasts formed through MSC differentiation. Intramembranous ossification may be initiated in response to injury and plays a crucial role in fracture healing, joint replacement, and dental implant surgery, and bone defects caused by trauma^43^. Despite researchers examining a range of factors such as growth factors and biomaterial scaffolds to enhance intramembranous ossification for bone regeneration therapies, difficulty in establishing standard treatment emphasizes the critical role of personalized medicine in bone regeneration^44^. The capacity for bone healing varies among individuals, and the related factors include age, health condition, and bone quality^45^. For example, impairments in angiogenesis have been observed during the healing of fractures in the elderly. Moreover, there is a notable decline in the quality and quantity of osteochondral cells^46^. Obesity causes a delay in the healing of the bone fracture^47^. Furthermore, bone defects may occur due to various factors such as trauma, infections, surgical removal of tumors, and congenital anomalies. Each type of defect presents unique challenges in terms of size, location, and surrounding tissue conditions^48 49^. Traditional approaches to bone regeneration, such as autografts, allografts, and synthetic bone substitutes, may yield variable outcomes depending on patient characteristics and defect parameters.

Multiple cells are involved in the intramembranous ossification process. Through the comparison of cellular functions in our experiment, it has been determined that the biological processes within different cell types exhibit a remarkable conservation. The major biological processes in iMPCs and iECs are related to cell proliferation, such as “organelle fission” and “chromosome segregation”, whereas the major biological processes in iMØs include “regulation of immune effector progress” and “leukocyte mediated immunity”. This aligns with the notion that cellular functions are intricately linked to cell types. Through the comparison of bone regeneration across various groups, it was observed that the bone formation exhibited variations in accordance with different pathways. Utilizing single-cell RNA sequencing data, CellChat analysis assists in the identification and characterization of signaling pathways and ligands that are implicated in intercellular interactions. Detection of the MIF (Macrophage migration inhibitory) pathway in the SCVI481 and SCVI3C1 MPC groups indicates the presence of coordinated crosstalk between iMØs and iMPCs. The highly outgoing CXCL signaling pathway observed in SCVI481-ECs and SCVI3C1-ECs indicates a strong communication between iMØs and iECs. The function of a specific cell type has the potential to affect the entire system. For example, the outgoing of the Wnt pathway was prominently detected in the iPS12-MPC group, and the Wnt pathway is subsequently highly activated in the cocultured bone organoid system. Our findings also indicate that a single molecule may be produced by different cells. The TGFβ pathway was mainly outgoing from the iMPC in the SCVI480 and SCVI3C1 groups but mainly outgoing from iMØ in iPS12 and SCVI481 groups. The data was not detected in coculture and bulk RNAseq analyses. This highlights the importance of investigating the functions of individual cell types in tissue regeneration.

### Mimicking clinical pathology using 3D co-culture models

Incorporating monocytes or macrophages into 3D tissue systems has been attempted by different groups, resulting in controversial findings. Factors secreted by pro-inflammatory macrophages substantially increased MSC attachment and migration whereas those released by anti-inflammatory macrophages enhanced MSC osteogenic activity as well as cell migration^57^. Others suggested pro-inflammatory M1 macrophages promote osteogenesis by MSC^50^. The process of bone regeneration is complex and requires coordination between immune cells and other cells at the site of injury.

3D culture system encapsulated iPSC-derived cells from same donor resemble tissue structure and functions. Verification of the intramembranous ossification in different donors using iPSC-derived cells is necessary to further elucidate its contribution in development, disease progressing. An inflammatory response occurs immediately following bone fracture. MØ are important in the protection of tissues from exogenous pathogens, and in tissue regeneration, and osteogenesis. MØs modulate the process of osteogenic differentiation during inflammation. Generally, naïve MØ will polarize to pro- or anti-inflammatory phenotypes which show different characteristics and modulate osteogenesis. M1 MØs are recognized to secrete pro-inflammatory cytokines and enhance osteoclastic activities, although some studies reported that M1 MØs helps enhance osteogenesis. Possible explanations include activation of the cyclooxygenase-2 (COX-2)- prostaglandin E2 (PGE2) pathway^50^. M2 MØs produce anti-inflammatory factors that promote tissue repairing and bone formation^51 52^. The crosstalk between MSCs and MØs is crucial for bone regeneration, and strategies targeting modulate macrophage phenotype have be explored. Inflammation plays a vital role in initiating the process of bone healing, but excessive acute or chronic inflammation can raise the risk of impaired bone healing. The expression levels of proinflammatory cytokines IL-6, MCP-1, and IL-8 were found to be significantly elevated across all our experimental groups, suggesting the presence of an initial inflammatory phase. Interestingly, the iPS12 group exhibited lower levels of IL-6 and MCP-1 compared to the other groups. TNFα and IL1Ra demonstrate elevated levels of expression in both the control group and the SVCI3C1 group. The pro-inflammatory cytokine TNFα has a complex role during bone healing^53 54^. TNFα-receptor-deficient mice showed delayed endochondral and intramembranous bone formation. IL-10, a cytokine associated with the acceleration of bone formation^55 56^, exhibits high expression levels in both the control group and the SVCI3C1 group. Our results highlight the importance of crosstalk between macrophages and MSCs during osteogenesis in 3D cultures.

Various growth factors play a role in the development of blood vessels, each with distinct effects on different molecules. FGF-2 induces blood vessels lacked vascular fenestrations and showed only little leakage of ferritin. VEGF-C induces approximately equal amounts of blood and lymphatic capillaries with endothelial fenestrations present only on blood capillaries, mediating a medium level of ferritin leakage into the perivascular space^58^. In addition to promoting angiogenesis, VEGF-C promotes the osteogenic differentiation and mineralization of MSCs^59^. Angiopoietin-2 stimulates the osteogenic differentiation of bone marrow stem cells^60^. Angiopoietin 2 promotes angiogenesis in tissue-engineered bone and improves repair of bone defects^61^. In response to inflammation, numerous cytokines are activated in tandem to regulate angiogenesis. MSCs also secrete the necessary factors for effective angiogenesis^62^. Our results showed that MØ increased the expression of VEGF-C in the control group, iPS12, and SCVI480 groups but reduced the level of VEGF-C in the SCVI3C1 group. The insulin level was elevated by iMØ, however, insulin did not have any impact on the primary control group. Osteogenesis is a complex process that is regulated by MØs and ECs. MSCs regulate the activity of ECs and other cells by releasing angiogenic factors. These factors may potentially modulate intramembranous osteogenesis.

The limitations of this study include the fact that only four lines of iPSCs were used to verify the generation of functional cells. Two iPSC cell lines (iPS12 and SCVI480) were from healthy donors, and two (SCVI481 and SCVI3C1) were from patients diagnosed with cardiovascular disease. Cell characteristics may change due to underlying medical comorbidities, medications, and the culture conditions^63^. Another limitation is related to immune regulation of biological processes; immune cells such as T cells and others will be involved in the crosstalk that modulates in tissue regeneration. We realize that cells derived from different iPSCs may lead to inconsistent experimental results, although the cells expressed comparable surface markers.

## Conclusion

The utilization of iPSC-based platforms has significantly advanced the progress in creating models of human diseases, autologous cell-based therapy, and drug discovery. While iPSC-derived cells display resemblances to primary cells, they also possess distinct characteristics. Cells obtained from different iPSC cell lines exhibited similar surface markers and functions. However, there was variation in the differentiation potential of iMPCs and the cytokine expression levels of iMØ. The bone organoid utilizing different iPSC-derived cells demonstrated distinct characteristics, and these variances have implications that extend to subsequent stages of tissue regeneration and disease modeling. Investigating personalized mechanisms in bone regeneration for the treatment of bone defects involves understanding the individualized factors that influence the healing process and tailoring treatment strategies accordingly.

## Materials and Methods

### Cell culture

iPS12 was obtained from Alstem Cell Advancements, which was derived from bone marrow MSCs. SCVI480, SCVI73, and SCVI481 cell lines were obtained from Joseph C. Wu, MD, Ph.D. from the Cardiovascular Institute, Stanford University (funded by NHLBI BhiPSC-CVD 75N9202D00019). The donor tissue is peripheral blood mononuclear cells. SCVI480 was derived from a healthy donor, SCVI73 was derived from a patient with arrhythmogenic right ventricular dysplasia, and SCVI481 was derived from a patient diagnosed with hypertrophic cardiomyopathy. To expand iPSCs, cells were cultured on a vitronectin XF (Stemcell Technologies, Vancouver, CA) coated plate, and fed with TeSR1 medium (Stemcell Technologies, Vancouver, CA). The medium was changed daily.

### iPSC differentiation

iPSC cell information is summarized in Table 1. Only iPS12 cell line was generated from bone marrow MSCs collected from a healthy donor, while the other 3 iPSC cell lines were obtained from peripheral blood mononuclear cells (PBMCs) from one healthy and two diseased donors.

The iPSCs were differentiated to iMPCs using the STEMdiff^™^ Mesenchymal Progenitor Kit (Stem Cell Technologies) according to the manufacturer’s protocol. Briefly, iPSCs were seeded at a density of 5000 to 10,000 cells/cm2 and maintained in mTeSR^™^1 medium supplied with 10 μM Y-27632. Cells were expanded by replacing medium to STEMdiffTMACF Mesenchymal Induction Medium for 3 days. Then, medium was changed to MesenCultTM-ACF Plus Medium (Stemcell Technologies). To passage the differentiated cells, pre-coated plates with Animal Component-Free Cell Attachment Substrate, and the cells were maintained in MesenCultTM-ACF Plus Medium.

To obtain iPSC-derived endothelial cells, iPSCs were cultured in Mesoderm Induction medium for 4 days first. Afterwards, culture medium was changed to endothelial differentiation medium (EGM2 medium (Lonza) supply with 50 ng/mL VEGF, 25 ng/mL FGF, and 10 μM SB431542 (Selleckchem)) for another 4 days. Then, CD34 microbeads was employed to sort iPSC-derived endothelial cells.

### iMSC differentiation

iMSCs were differentiated into osteoblasts by culturing in osteogenic medium (DMEM supplemented with 10% FBS, 1% antibiotic-antimycotic, 0.01M β-glycerophosphate, 50 mM ascorbic acid, 100 ng/mL bone morphogenetic protein 7 (BMP7), 10 nM Vitamin D3, and 100 nM dexamethasone) for 4 weeks (dexamethasone and BMP7 were removed from the medium for the last 2 weeks). To obtain chondrocytes, iMPCs were cultured in StemPro^™^ chondrogenesis differentiation kit (Thermofisher A1007101) for 4 weeks. StemPro^™^ adipogenesis differentiation kit (Thermofisher A1007001) was applied to differentiate iMPCs to adipocytes.

### qPCR

qPCR was applied to compare the related gene expression. Briefly, mRNA was extracted using TRIZOL and reversed to mRNA. To quantify the expression level, Taqman gene expression primers were applied, including GAPDH (Hs02786624_g1), SOX 9 (Hs00165814_m1), Collagen X (Hs00166657_m1), COMP (Hs00164359_m1), CEBPA (Hs00269972_s1), PLIN1 (Hs00160173_m1), LPL (Hs00173425_m1), PPARG (Hs01115513_m1), RUNX2 (Hs01047973_m1), and SPP1 (Hs00234160_m1).

### iEC permeability assay

The endothelial cell barrier integrity was determined with the transwell insert permeability assay. Briefly, 20,000 ECs were grown on a Transwell insert (with polycarbonate finish, 0.4 μm pore; Corning Costar, USA) until confluent. The cell monolayers were exposed to FITC–dextran (5 μg/ml) for 1 hour. The fluorescent intensity at the lower compartment of the transwell was measured in a microplate reader at excitation/emission wavelength of 485/538 nm. Normalizing the fluorescence signals to the standard curve gives the degree of leakiness.

### Fluorescent microscopy and immunostaining

The cells were fixed in 4% paraformaldehyde (PFA). iMPCs-derived osteocytes were stained with 40 mM Alizarin Red and alkaline phosphatase (1-Step^™^ NBT/BCIP Substrate Solution) (Thermofisher 34042). iMPCs-derived chondrocytes were stained with Alcian Blue (pH 2.5) Stain Kit (Vector Laboratories, H-3501). iMPCs-derived adipocytes were stained with Oil Red O Stain Kit (Abcam ab150678).

iMPCs were trypsinized, washed and incubated with the following antibodies: FITC Mouse Anti-Human CD31, CD34, CD45, CD73, and CD90 (BD Biosciences, Franklin Lakes, NJ). Cells were then analyzed by flow cytometry (BD FACS AriaTM II cell sorter; BD Biosciences) to assess the expression of these cell surface epitopes.

### Staining of 3D scaffold

Scaffolds were fixed in 4% paraformaldehyde (PFA) solution, dehydrated by 30% sucrose, and embedded in optimal cutting temperature (OCT). Then, the embedded samples were sectioned at 10 μm thickness using microtome.

### scRNA-seq analysis

Seurat^64^ (version 4.0.5) in R software was used for chondrocyte scRNA-seq data processing. Cells with great proportion of mitochondrial gene expression were removed. The cell cluster was manually annotated using the identified cell type marker genes. Utilizing the CellChat package in R, cellular communication was analyzed and quantified by merging single-cell expression profiles with prior knowledge of signaling ligands, receptors, and their cofactors.

### RNA-seq analysis

Gene Ontology (GO) is used to identify enriched functions of genes in three independent categories: Biological process (BP), molecular function (MF) and cellular component (CC). Kyoto Encyclopedia of Genes and Genomes (KEGG) was used to identify relevant pathways for genes. Ingenuity Pathway Analysis (IPA) server (Qiagen, Redwood City, CA) was used to perform canonical pathway and upstream regulators analysis.

## Figures and Tables

**Fig 1.**
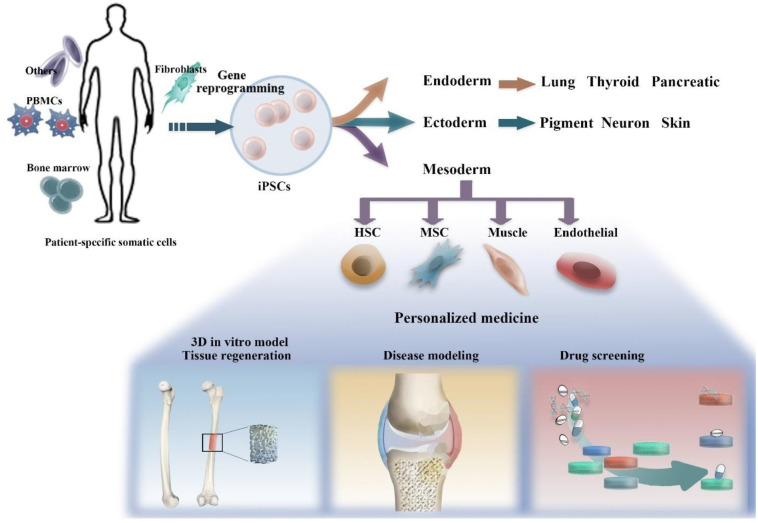
Illustration of generating iPSCs from a donor’s somatic cells, and the differentiation of iPSCs to other mature/immature cell types. iPSC-derived cells can serve as valuable tools for modeling bone regeneration and diseases related to joints. Furthermore, these models can be utilized for the purpose of personalized drug screening.

**Table 1:** 

	Donor Tissue	Gender	Age	Disease
iPS12	Mesenchymal stromal cells	Female	-	Healthy donor
SCVI480	Peripheral Blood Mononuclear Cells	Female	18	Healthy donor
SCVI481	Peripheral Blood Mononuclear Cells	Male	23	Hypertrophic cardiomyopathy
SCVI73	Peripheral Blood Mononuclear Cells	Male	65	Arrhythmogemc right ventricular dysplasia
